# Blocking and re-arrangement of pots in greenhouse experiments: which approach is more effective?

**DOI:** 10.1186/s13007-019-0527-4

**Published:** 2019-11-27

**Authors:** Jens Hartung, Juliane Wagener, Reiner Ruser, Hans-Peter Piepho

**Affiliations:** 1 Institute of Crop Science, Biostatistics Unit, University of Hohenheim, Stuttgart, Germany; 2 Institute of Crop Science, Department Fertilization and Soil Matter Dynamics, University of Hohenheim, Stuttgart, Germany

**Keywords:** Re-randomization, Re-arrangement, Experimental design, Greenhouse experiment, Rotation, Relocation

## Abstract

**Background:**

Observations measured in field and greenhouse experiments always contain errors. These errors can arise from measurement error, local or positional conditions of the experimental units, or from the randomization of experimental units. In statistical analysis errors can be modelled as independent effects or as spatially correlated effects with an appropriate variance–covariance structure. Using a suitable experimental design, a part of the variance can be captured through blocking of the experimental units. If experimental units (e.g. pots within a greenhouse) are mobile, they can be re-arranged during the experiment. This re-arrangement enables a separation of variation due to time-invariant position effects and variation due to the experimental units. If re-arrangement is successful, the time-invariant positional effect can average out for experimental units moved between different positions during the experiment. While re-arrangement is commonly done in greenhouse experiments, data to quantify its usefulness is limited.

**Results:**

A uniformity greenhouse experiment with barley (*Hordeum vulgare* L.) to compare re-arrangement of pots with a range of designs under fixed-position arrangement showed that both methods can reduce the residual variance and the average standard error of a difference. All designs with fixed-position arrangement, which accounted for the known north–south gradient in the greenhouse, outperformed re-arrangement. An α-design with block size four performed best across seven plant growth traits.

**Conclusion:**

Blocking with a fixed-position arrangement was more efficient in improving precision of greenhouse experiments than re-arrangement of pots and hence can be recommended for comparable greenhouse experiments.

## Background

Observations made in experiments deviate from their expected values. These deviations are called errors. They occur, e.g., due to measurement errors, differences between experimental units and differences in environmental conditions experimental units are exposed to [1]. The experimental unit is the smallest unit within an experiment to which a treatment is randomly assigned [2]. Even though errors can arise for different reasons, separating or distinguishing different error effects is not always possible. For example, in a greenhouse evaluation trial with different crop cultivars tested for their yield performance in small pots, the observed yield in a pot deviates randomly from the expected yield of the cultivar grown. As cultivars are randomly assigned to pots, the pot is the randomization unit. If pots are located at fixed positions during the experiment, both, the cultivar and the soil of a pot, remains at the same position throughout the experiment. Thus, there are two sources of error effects: the effect of the soil of a pot and the effect of the environment condition the pot is exposed to. These effects cannot be separated from each other. Pot or residual effects and environmental effects are completely confounded. A single (total) effect summarizing both error effects can be fitted in a model for this type of data. Error effects are usually small in greenhouse and climate chamber experiments as compared to error effects in field experiments, because environmental conditions are more controlled. Nevertheless, variation within a greenhouse is also common [3]. Furthermore, period-to-period variation from using the same greenhouse in a temporal sequence of experiments and greenhouse-to-greenhouse variation are known to be relevant in greenhouse experiments [4–7].

One option to account for this variation is to conduct the experiment according to a blocked experimental design. The simplest experimental design with blocks is a randomized complete block design (RCBD), where treatments are replicated once within each block. If blocks are orthogonal to a gradient, the variation due to this gradient can be accounted for by a block effect in the analysis. In this case, blocks capture a part of the error, thus reducing the residual variance component (VC), which only amounts to the variance of error effects within blocks. If the number of treatments used in a RCBD gets large, experimental designs with incomplete but smaller blocks are preferred, e.g. α-designs. The blocks in these designs are incomplete in the sense that not each treatment is replicated in each block. If there are gradients in more than one direction, the use of row–column designs with blocks in rows and in columns can be used. If incomplete blocks can be grouped into complete blocks, the design is called resolvable. If a blocked experimental design is used for an experiment and the blocks capture a part of the variance, an analysis accounting for the experimental design can be more efficient compared to an analysis not accounting for the design [8, 9]. The gain in efficiency results from the possibility of removing the variance between blocks from the total variance, making the residual variance smaller. However, this efficiency gain comes at the cost of some degrees of freedom and a more restricted randomization. The use of experimental designs in planning and analyzing experiments is well known to be efficient (e.g. [10–12] and references within). Note that a gain in efficiency of a design means a reduction in the variance for the difference between two treatment means compared to a contending design, such as a completely randomized design [10]. A range of different resolvable and non-resolvable designs with complete or incomplete blocks in one or two directions and varying block sizes were suggested [1, 13–17]. These experimental designs differ in their ability to account for spatial variation and gradients within the experimental area. An alternative to randomization-based modelling of the total variance is the separation into global, extraneous and natural variation using spatial models [18]. In this case, splines, random effects for row, and column, and a spatial correlation of error effects are fitted to data. For spatially correlated residual effects a linear or autoregressive variance–covariance structure can be fitted [19]. Other functional variance-to-distance relationships in one or two directions can be fitted, such as isotropic or anisotropic spherical, Gaussian or exponential models [20]. All these methods can be applied to data from greenhouse and climate chamber experiments as well as to field experiments, where experimental units have a fixed position throughout the experiment.

In field experiments, experimental units are mostly plots, which are immovable. However, experimental units in greenhouse and climate chamber experiments, which are usually pots, can either be located at fixed-positions [21] or moved around within the experimental area during the experiment [7, 22–26]. In the latter case, experimental units are exposed to different positions, and therefore potentially to different environmental conditions within the experiment. This pattern allows separating of the effects attached to the pot (thus the pot or residual effect) and the time-invariant positional effects. If re-arrangement of experimental units is repeated over time so that positional effects equally affect all experimental units, positional effects can be hoped to average out. In this case, at the cost of the work load associated with the movement of experimental units, re-arrangement provides an opportunity to remove positional effects from the total variance [1, 3]. In the most favourable case, positional effects are constant over time and plant growth stages. Furthermore, experimental units should be exposed to all positional effects for the same amount of time [3]. In practice, re-arrangement of experimental units is often done in some systematic order, by rotating experimental units [27–29] or groups of experimental units [3, 7].

For greenhouse experiments, where experimental units are portable, there exist two options for making experiments more efficient: (i) the use of a blocked design for both performing an experiment and analyzing its data, and (ii) the use of a re-arrangement of experimental units for performing the experiment and analyzing its data. While the efficiency of experimental designs was shown theoretically [8, 9, 30] and empirically [10–12] a long time ago, the efficiency of re-arrangement of experimental units has not been evaluated thoroughly [31]. An enquirer in the journal *Biometrics* in 1957 [1] was the first to raise the question of whether it is proper or improper to re-arrange experimental units in greenhouse experiment. Kempthorne argued in his answer that the efficiency depends on the ratio of the variance of positional and pot effects. Little work on this problem seems to have been done since. We are aware of only five papers on the empirical evaluation of a re-arrangement of experimental units: [27] showed that rotating pots on a rotating table reduces the residual VC compared to the variance estimated from a normal, fixed-position table. But unfortunately, pots on the rotated table and the fixed-position table were arranged differently with pots having more space between each other on the rotated table. Thus, the effect of rotating pots was confounded with the varying space between pots (and hence varying neighboring effects). Lazarovitch et al. [22] performed an unreplicated shading experiment showing a decreased standard error for re-arranged pots compared to a fixed-position layout using a completely randomized design. Brien et al. [3] used a two-phase experiment to study the effect of four different arrangements in the second phase of a greenhouse experiment on growth traits in wheat. The authors concluded that re-arrangement of experimental units can reduce errors for total plant area, but an appropriate experimental design and analysis will achieve the same result more easily and reliably. Unfortunately, this study lacks true replicates for the arrangement. This can be problematic when comparing re-arrangement and fixed-position arrangement, as zones within the greenhouse allocated to different arrangements vary according to their environmental conditions. Two further experiments, with small-scale and large-scale phenotyping platforms, were performed in [7]. In the small-scale phenotyping platform, re-arrangement and fixed-position arrangement were tested in the same experiment but in separate regions within the room (on the rotating line and aside the line). The large-scale experiment compared both arrangements in separate experiments. Furthermore, in the large-scale experiment the 12 control pots were allocated to the diagonal of the chamber with one pot per line. Tisné et al. [28] ran separate experiments with continuously rotating of pots and without rotating of pots. The authors found that evaporation was more homogeneous with rotating. It is noted that once again in the fixed-position arrangement data were analyzed assuming a completely randomized design and thus with a model which cannot account for spatial variation via block effects. While the authors of these five papers showed that re-arrangement of experimental units reduced residual variance, their experiments do not permit conclusions about the efficiency of re-arrangement of experimental units compared to using a blocked experimental design. The limitations in the data to answer the question of whether or not to re-arrange pots in greenhouse experiments is slightly surprising, as most of the researchers working on the design of greenhouse experiments have a decided answer to this question, either against the use of re-arrangement [3, 23, 32–34] or in favor to its use [22, 24, 26].

The aim of the current study is therefore to perform a randomized and replicated experiment to give an empirical answer to the question of whether re-arrangement of experimental units or the use of a blocked experimental design in planning and analyzing the data is the preferred method for efficient greenhouse experiments. Barley (*Hordeum vulgare* L.) was used here as a model plant for cereals.

## Results

Ignoring the superimposed treatment design and analyzing simply the observed data demonstrates that re-arrangement of pots (which are the experimental unit in our experiment) tends to reduce the error VC. The results given in Table 1 are based on model (1), and they show that the ratio of the fixed-position error VC divided by the re-arrangement error VC for the seven traits varies between 0.39 and 2.35 with an average of 1.09 across traits. Fitting random positional effects (model 6) resulted in a zero VC estimate in six out of seven traits. For the trait ‘C content’ the VC is different from zero and the Akaike information criterion (AIC) increases by 1.9. Thus, from the analysis of the observed data, fixed-position pots tend to have a slightly larger error VC. Modelling position effects under the assumption of time-invariant position effects was not successful. Furthermore, plants in pots on the northern and southern end of the experimental area were visually more homogeneous under re-arrangement compared to fixed-position arrangement.

**Table 1:** Evaluation criteria (error VC and their ratio) for seven traits and across traits under fixed-position design and re-arrangement

Trait	Error variance component		
	Fixed-position	Re-arrangement	Ratio (fixed-position/re-arrangement)
Fresh weight	34.9211	14.8595	2.35
Dry weight	0.2532	0.1905	1.33
SPAD day 30	14.7888	19.4623	0.76
SPAD day 36	6.1816	8.1668	0.76
C content	0.0687	0.1748	0.39
N content	0.1068	0.1058	0.99
C to N ratio	0.0111	0.0105	1.06
Average across traits			1.09

*SPAD* single photon avalanche diode

For results of different experimental designs, estimates were averaged across randomizations. Afterwards, the ratios of the error VC and the standard error of a treatment difference (s.e.d.) for each trait were calculated (Additional file 1: Tables S1, S2). These ratios were then averaged across traits (Table 2 or the last column in Additional file 1: Tables S1, S2). For a randomized complete block design (RCBD), the ratio of the error VC across randomizations varies between 0.39 and 2.40 with an average of 1.09 across traits. These values are similar to the values estimated from observed data without superimposed treatments, as both based on the same block structure. The ratio of average s.e.d. varies between 0.6 and 1.54 with an average of 1.02. For the analysis of designs 1B, 1M and 1N, the average ratio of s.e.d. and error VC across traits were close to, or equal to one. For all other analyses of fixed-position data (using designs 1C-1L), the average ratios of s.e.d. and VC across traits were lower than one. This means that across randomizations and traits all block designs were as good as re-arrangement. Indeed, most of the designs outperformed re-arrangement (Table 2).

**Table 2:** Average ratio of evaluation criteria [error variance component (VC) and standard error of a treatment difference (s.e.d.)] for a given experimental design compared to the evaluation criteria using re-arrangement of pots across seven traits

Design (number of blocks × block size)	Efficiency factor [2]	Average ratio^b^ of	
		error VC across traits	s.e.d. across traits
RCBD			
1 × 20 (1A)	1	1.09	1.00
α-design			
2 × 10 (1B)	0.90	1.05	1.02
2 × 10 (1C)	0.90	0.89	0.96
4 × 5 (1D)	0.75	0.81	0.97
4 × 5 (1E)	0.75	0.78	0.94
4 × 5 (1F)	0.75	0.75	0.94
5 × 5 (1G)	0.68	0.70	0.94
5 × 5 (1H)	0.68	0.78	0.98
5 × 4 (1I)	0.68	0.71	0.94
5 × 4 (1J)	0.68	0.63	0.91
10 × 2 (1K)	0.14	0.62	0.96
10 × 2 (1L)	0.14	0.66	0.98
Row–column design			
2 × 10 (1M)	–	0.59	1.02
2 × 10 (1N)	0.63	0.60	1.02
α-design (1B) with spatial error structure^a^	0.90	–	1.00

200 randomizations for each trait and design were performed and results are based on analyses reaching convergence

*RCBD* randomized complete block design, *SPAD* single photon avalanche diode

**Fig. 1:**
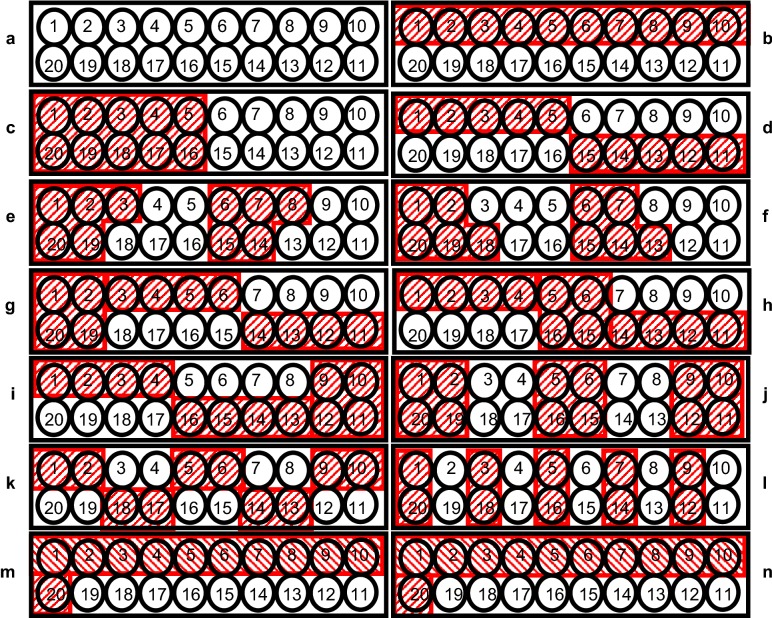
Assignment of pots on a table to blocks of different designs. In all cases, a design with 20 treatments was superimposed onto the 20 pots per table. **a** Randomized complete block design (RCBD) with 20 pots per complete block. **b**, **c** α-design with two incomplete blocks along rows or columns. **d**–**f** α-designs with four incomplete blocks. **g**–**j** α-design with five incomplete blocks each with four pots. **k**, **l** α-design with ten blocks of size two. **m**, **n** resolvable/non-resolvable row–column design with two row and ten column blocks per table

^a^Nugget variance plus a first-order autoregressive error structure within a block of ten pots arranged in a row on the table (Fig. 1b)

^b^Ratio for all designs of either the VC or s.e.d. for fixed-position arrangement to the VC or s.e.d. under re-arrangement, respectively

It should also be noted that error VC decrease with increasing number of blocks, but at the cost of losing error degrees of freedom. There is a trade-off between smaller VC and decreasing efficiency factor [2], so VCs alone are less informative (Table 2) and the s.e.d. is more meaningful. From s.e.d. results of our simulation with 20 superimposed treatments, a block size of four pots seems to be optimal with an arrangement visualized in Fig. 1j. Incomplete blocks and spatial error structure improve model fit according to AIC on average across randomizations. Furthermore, AIC decreases in about two out of three randomizations for all these designs (results are not shown).

## Discussion

### Results in other papers

Re-arrangement of pots can average out positional effects, and therefore reduce the residual or pot-to-pot variance if pots are equalized in their exposure to microclimates or environmental condition present in the experiment [1, 3, 26]. This would mean that the accumulated positional effects are the same for each pot. The simplest case when re-arrangement of pots successfully averages out positional effects is as follows: (i) all pots were located in each position an equal amount of time, and (ii) all positional effects were constant over time and over growing stages. Note that there could be other, more relaxed conditions still fulfilling the requirement that pots are subject to the same positional effects over time. But for these cases, both the proof that the sum of positional effects is indeed constant, and finding a design for re-arranging pots gets more difficult. If these conditions are fulfilled, positional effects are successfully removed from errors [1]. Otherwise, positional effects can be reduced only incompletely. In our experiment, pots were located on only 13 out of 20 positions for a varying time between one and seven days per position. Brien et al. [3] argued that such a haphazard re-arrangement of pots during an experiment will not equalize their exposure to microclimate. Therefore, we tried to model the 20 position effects being spread to 13 pots each (model 6). This model assumes time-invariant and growth-stage-invariant positional effects. Results showed no advantage in model fit. Thus, positional effects were either time dependent or they were small enough so as not to explain a sufficient amount of variance.

Error effects can be assumed as independent or as spatially correlated [18]. Two approaches are possible when errors are assumed to be spatially correlated and the experimental units were randomized using a blocked experimental design. First, block effects are included in the model and errors within a block were considered as spatially correlated. Spatial correlation is taken as an add-on to the randomization-based analysis [35]. Second, fixed-position data can be analyzed with a spatial error that applies to the whole experimental area and the fitting of blocks effects depends on the evidence for those block effects occurring in the experiment [18]. Note that we used only the first approach here. Furthermore, only error effects within a row were assumed as independent or as spatially correlated with an additional first-order autoregressive variance–covariance structure in the analysis. In this special case, results showed no advantage of including a spatial correlation. This is in line with results found in, e.g., [35].

In most greenhouse experiments, the heterogeneity of environmental conditions is not measured. If such information is available, there are two options. First, the information can be used to optimize re-arrangement of pots. Assuming that temperature and radiation are the most important variables for heterogeneous microclimates within the green-house, [26] showed that optimized re-arrangement of pots can reduce accumulated positional effects by 90%. Second, for a fixed-position arrangement, this information can be used as a covariate [7, 36].

The performed experiment estimates the effect of re-arrangement versus fixed-position arrangement in a small randomized and replicated greenhouse experiment. The seven traits considered were chosen because they are commonly measured in experiments performed in this greenhouse [37, 38]. These traits are correlated, and so they are not independent. Nevertheless, we believe that the results found will hold more broadly for greenhouse and climate chamber experiments having smaller positional effects compared to the experiment considered as both arrangements get more efficient with larger positional effects. Note that the conditions during the experiment were chosen so that the potential advantage of re-arrangement of pots is maximized. To do so, a non-heated greenhouse with a large and well-known gradient in north–south direction was used and tables within the experiment were rotated to maximize positional effects. The gradient is mainly caused by differences in light and temperature, as the greenhouse has glass in the north and mesh in the south. Temperature is the most variable condition in non-heated greenhouses [15, 39]. Its influence on growth is maximized if temperature is critical for growth, therefore the experiment was performed in spring [40–42]. The tables were arranged within the greenhouse so that the expected gradient is parallel to the long side of each table (further information about the greenhouse can be found in Additional file 1: Figures S1, S2).

The chosen experimental settings were successful in the sense that differences between pots were visibly reduced under re-arrangement compared to fixed-positioning. Pots located at both sides of the table at the start of the experiment, showed similar growth status and plant height under re-arrangement and reduced growth for pot located at the southern end under fixed-positioning. Furthermore, positional effects were large enough to slightly reduce the average error VC estimate under re-arrangement compared to fixed-position arrangement using model (2). Also, the drop of residual VC using incomplete blocks or row and column effects and the improved model fit when including these effects show the existence of different positional effects [3]. In our experiment, the average residual VC (across traits and randomizations) was reduced by 5% to 50% if incomplete blocks were fitted. This reduction is similar to the reduction of 36% observed in cultivar evaluation field trials [43] and the 36% to 52% reduction observed by Lee and Rawlings [4]. While positional effects did exist, re-arrangement of pots did not reduce the s.e.d. of treatment differences as compared to a superimposed incomplete block design. While designs with blocks in west–east direction (1B, N and M) are as good as re-arrangement, designs with blocks in north–south direction outperformed re-arrangement. This is in accordance with [3] and theoretical arguments given by [15]. However, [27] showed the usefulness of re-arrangement. But note that in their experiment, the re-arrangement effect was confounded with the effect of larger pot space.

### Convergence problems due to lack of sufficient error degree of freedom

The arrangement methods were replicated twice, where replicates corresponding to tables used in the experiment. 40 pots, arranged on two tables each with two rows of ten pots, were measured for each arrangement. For the fixed-position arrangement, a row–column design was assumed, where 20 treatments are virtually allocated to two rows and ten columns per table. The model accounting for this row–column design is saturated. For the α-design with ten incomplete blocks per table, only one error degree of freedom remained. For both designs, convergence problems using the default model fitting conditions (default starting values, 50 iterations) occurred in 1 to 5% of the randomizations. Using starting values from parameter estimates of other randomizations was rarely a successful strategy to get convergence. Results from randomizations without convergence were dropped. Note, that for a lower number of virtually superimposed treatments, the error degrees of freedom increase, which appears to eliminate convergence problems.

### Application of re-arrangement of pots and experimental designs to experiments performed on phenotyping platforms

Re-arrangement of pots is used in the context of vertical farming [6], but the highest relevance is its application in phenotyping platforms [28, 44, 45]. The latter are intensively investigated as one option for high-throughput phenotyping methods, e.g. [44–51]. The reason for the high relevance is that phenotyping platforms allow an easy use of re-arrangement, as pots are moved for watering or phenotyping anyway. Therefore, there is nearly no additional workload [24]. Thus, efficient high-throughput phenotyping raises questions about the integration of optimal experimental design [52], and the possible re-arrangement of pots. Depending on the phenotyping platform, the number of choices to re-arrange pots differ. For example, the IPK Lemna Tec Scanalyzer system can automatically phenotype 384 carriers (384 pots or trays with several pots per tray). This forms a single series of carriers, which means that pots are always standing between the same two neighbors [7]. Also, weighing and watering is done in the same order. The HT automated non-invasive phenotyping (LemnaTec) system allows re-arranging single pots or group of pots. In a single cycle, where all pots are weighed and therefore moved once, re-arrangement of pots is restricted by the order in which pots are selected. Only pots at the end of lines can be selected and can be moved to the beginning of another line. This may cause restrictions in the re-arrangement options.

Brien et al. [3] reduced the south-north gradient within the greenhouse by rotating full lines of experimental units. Junker et al. [7] concluded that re-arrangement of groups of pots is sufficient for reducing or eliminating spatial variance between blocks. Thus, the objective of [3, 7] was to remove or reduce block effects. In our experiment, pots were re-arrange to reduce the residual VC under a RCBD. This was successful too, even if the use of a blocked design with (additional) incomplete blocks was more efficient, which was already expected from [3]. Thus, re-arrangement can be applied to pots or blocks within the experiment and therefore reduce the variance between pots or between blocks. For RCBD, the variance of blocks does not influence the precision of treatment differences. Note that for these designs blocks are orthogonal to treatments so treatment means require no adjustment for blocks and there is no inter-block information. For designs with incomplete blocks such as α-designs and augmented designs, the block variance estimate influences the amount of inter-block information to be recovered. Thus, a combination of an experimental design and a randomized re-arrangements of incomplete blocks can be an interesting option. Furthermore, if conditions within blocks of a blocked experimental design are not homogeneous, re-arrangement can be another promising option as it can reduce the residual VC within complete or incomplete blocks. Note that re-arrangement within blocks is likely to be ineffective when blocking is effective.

### Distinguishing between movement-effects and re-arrangement

There exists a well-documented movement-effect or thigmomorphogenic effect on plant growth [53], e.g. in tomato [54] and maize [7]. Greenhouse plants moved around showed reduced growth but increased stability and increased yield. The effect results from the movement of plants simulating injuries due to wind. As in our experiment plants in pots in both arrangements are equally moved around for watering, the movement effect should influence results equally. The only difference between the re-arrangement and the fixed-position arrangement in our experiment was that pots were allocated to varying or the same positions after watering.

## Conclusion

In our greenhouse experiment with barley, re-arrangement of pots within a replicate successfully reduced the average s.e.d. But any reasonable block structure outperformed re-arrangement of pots. Therefore, blocking with a fixed-position arrangement can be suggested for use in comparable greenhouse experiments.

## Methods

The aim of the current study is to perform a randomized and replicated greenhouse experiment to give an empirical answer to the questions of whether re-arrangement of pots or the use of experimental designs in performing the experiment and analyzing the data is the preferred method for efficient greenhouse experiments.

### Greenhouse experiment

The experiment was conducted in spring 2016 in a greenhouse of the University of Hohenheim. The non-heated greenhouse has a large known temperature gradient from the door at the northern side to the window to a mesh-protected area outside at the southern side (Additional file 1: Figures S1, S2). Windows at the southern side were opened during day time if temperatures were not too cold for barley growth. Another orthogonal environmental gradient cannot be precluded. Therefore, four tables were arranged in a 2-by-2 set-up, and arrangements were randomly assigned to the two diagonals. Thus, the arrangement methods were replicated twice, where replicates correspond to tables used in the experiment.

On each table, 20 Mitscherlich pots were allocated in a 2-by-10 grid with two rows and ten columns (Fig. 1, in which north is on the left). The 80 pots had a volume of 6.2 l each. They were filled with a mixture of 5 kg of sieved (<4 mm) top soil sampled from an arable Haplic Luvisol and quartz sand (0.6–1.2 mm) to a total weight of 7.45 kg. Soil texture of the top soil was composed of 3.5% sand, 65.8% silt and 30.7% clay. Organic C-content was 1.44% and the initial pH value in 10^–2^ M CaCl_2_ solution (soil:solution 1:2.5 by weight) was 6.8. The nutrients (0.5 g N, 1 g K, 0.75 g P, 0.5 g Mg in solution of 0.6 l water per pot) were added and mixed carefully with the soil-quartz mixture. In each pot, 30 summer barley seeds (variety: RGT Planet) were sown and covered with 1.5 cm quartz sand. Seeds were sown on March 9th 2016 (day 0). Germination started on March 14th (day 5). To equalize the number of seedlings per pot they were reduced to 21 on day 13. Furthermore, the number of plants were reduced to 20 on day 24.

During the growing period, each day a random sample of two to three pots per table were weighted to determine the average water loss. This measurement was used to determine the aliquot of water given to all pots to balance daily weight loss. Once a week, all pots were weighted individually and water was added to a given weight between 8.08 kg (after sowing) and 8.3 kg (after day 21). To avoid nitrogen deficiency, 0.25 g N as NH_4_NO_3_ was given to each pot on days 14 and 24. Chlorophyll content was measured by a single photon avalanche diode (SPAD, [55]) at days 30 and 36. Plants were harvested on day 37. All 20 plants from a pot were bulked to one sample. Fresh weights of the bulked samples were measured. Samples were dried at 60 °C to constant weight. Total C and N concentrations in dried plant material aliquots were determined using a total CN analyzer (Vario Max CN, Elementar Analysesysteme, Hanau).

In total, five values of the whole data set were detected as outliers via residual plots and plausibility checks; one SPAD value on day 36, two values of C and two values of N content. All outliers were detected under re-arrangement and were dropped from the analysis.

### Arrangement of pots on a table

Two different pot arrangements were compared. In the first arrangement, pots on tables with fixed-position arrangement were randomly allocated to positions on a table once and were not moved afterwards (except for weighing and watering, after which they were replaced to their fixed positions). Pots were returned to the same position after weighing. In the second arrangement, pots were rotated in the following way: on days 6, 7, 15, 22, 25, 30, and 33 the 20 pots on a table were cyclically moved clockwise direction to the next position. Additionally, re-arrangement tables were rotated by 180° on days 8, 17, 20, 26, 28, 30, 32, and 34. Therefore, each pot was placed at several positions during the experiment. For example, the pot originally located at position 1 was allocated to a total of 13 different positions during the experiment (positions 1–4, 6–8 and 13–18).

### Superimposed treatment allocation onto pots

As described previously, a uniformity trial per table was performed. Thus, all pots were treated equally, apart from the two different arrangements. In practice, pots in designed experiments are treated differently as the scientist is interested in comparing treatments, and treatments are allocated to pots (and not to tables within the greenhouse). Therefore, for analysis (and thus after performing the experiment) we superimposed a treatment factor onto the uniformity trial of each arrangement. The 20 levels of this treatment factor were allocated to the 20 pots per table. This merely involved assigning treatment labels to pots, but no treatment effects, meaning that the simulated effects of the superimposed treatments were zero. A range of different experimental designs were used to superimpose treatments onto pots. An RCBD with 20 treatments was used for both arrangements. Furthermore, for the fixed-position arrangement, α-designs and resolvable and non-resolvable row–column designs were used. In all designs, 20 treatment levels were assumed. The experimental designs used in the experiment are shown in Fig. 1a–n. 200 randomizations were used for each design. All randomizations for all designs were created using CycDesigN 5.1 [56].

### Data analysis

Ignoring the superimposed treatment designs, the following model was fitted to the uniformity data:

1 $${y}_{ijm}=\mu +{\tau }_{i}+{t}_{ij}+{e}_{ijm},$$

where ${y}_{ijm}$ is the response measured on pot *m* of table *j* treated with the *i*th arrangement, $\mu$ is the intercept, ${\tau }_{i}$ is the fixed effect of the *i*th arrangement, ${t}_{ij}$ is the random effect of the *j*th table treated with arrangement *i*, and ${e}_{ijm}$ is the error effect of ${y}_{ijm}$ with arrangement-specific variance $\left({e}_{ijm} \sim N\left(0,{e}_{i}^{2}\right)\right)$. Note that the error variance for re-arrangement corresponds to the sum of the residual variance and the variance of positional effects accumulated during the experiment. For fixed-position arrangement it corresponds to the residual variance plus the variance of positional effects.

For analyzing data with superimposed treatment designs, a split-plot model with main plot factor ‘arrangement’ (with levels fixed-position and re-arrangement) and sub-plot factor ‘treatment’ was used. Recall that the assignment of 20 virtual treatments follows a RCBD for the re-arrangement or a range of different designs for the fixed-position arrangement. A design-specific model with different random effects for incomplete block, row or column effects was fitted. For the RCBD, the following model was fitted:

2 $${y}_{ijkm}=\mu +{\tau }_{i}+{\varphi }_{ik}+{t}_{ij}+{e}_{ijkm},$$

where ${y}_{ijkm}$ is the response measured on pot *m* treated with the *i*th arrangement and the *k*th treatment on the *j*th table. Note that observations are indexed by *m* and *k* even if on a table *ij* only one treatment is superposed on one pot. Therefore, one of the two indices would be sufficient, but both are kept here for consistency of notation. ${\varphi }_{ik}$ is the effect of the *k*th treatment at the *i*th arrangement and all other effects are analogous to (1). Further note that ${t}_{ij}$ is the random effect of the *j*th table with *i*th arrangement and therefore the main plot error. Without loss of generality, superimposed treatments differ between arrangements. Thus, the model includes no main effect for treatment. When treatments were arranged according to an α-design, the model was extended to

3 $${y}_{ijklm}=\mu +{\tau }_{i}+{\varphi }_{ik}+{t}_{ij}+{b}_{ijl}+{e}_{ijklm},$$

where ${b}_{ijl}$ is the random effect of the *l*th incomplete block within the *ij*th table. All other effects are analogous to (1). Note that ${b}_{ijl}$ was only fitted to fixed-position data. A dummy variable to block out data of re-arrangement from estimation of block effects was used for this purpose [57], but is not shown in the model description for simplicity. For the row–column designs, the model is extended to

4 $${y}_{ijklmo}=\mu +{\tau }_{i}+{\varphi }_{ik}+{t}_{ij}+{r}_{ijl}+{c}_{ijo}+{e}_{ijklmo},$$

where ${r}_{ijl}$ and ${c}_{ijo}$ are the random *l*th row and *o*th column effects on the *ij*th table, respectively. All other effects are analogous to (1). As an alternative to including further block effects, a first-order autoregressive error structure was assumed in (2), and a block size of ten along the columns (model 5). Again, a dummy variable was used to fit effects of rows and columns in (4) and columns and spatial correlated errors in (5) under fixed-position arrangement only.

Furthermore (1) is extended by adding a positional effect for re-arranged data only. This model assumes that the *p*th position effect on the *ij*th table is constant over time. As re-arranged pots are subject to 13 different position effects over time, dummy variables were defined for each position. The complete position ${p}_{ijp}$ effect is divided among all pots allocated at this position. This division was assumed to be proportional to the time a pot was allocated to the corresponding position. The extended model (model 6) of (1) is:

5 $${y}_{ijm}=\mu +{t}_{ij}+{p}_{ijm}+{\tau }_{i}+{e}_{ijm} \quad {\text{with}} \; {p}_{ijm}=\frac{\sum_{p=1}^{20}{p}_{ijp}\cdot {t}_{ijmp}}{\sum_{p=1}^{20}{t}_{ijmp}},$$

where ${p}_{ijm}$ is the sum of all products of position effects ${p}_{ijp}$ and the time pot *m* was allocated on position *p* on table *ij* (${t}_{ijmp}$). Note that the position effect in the fixed-position arrangement is confounded with the error effect, thus it can be separated from the error effect for the re-arranged pots only. Again, a dummy variable was used to make sure position effects are only estimated for rearranged-position data but was not shown in the model description to simplify the presentation. As position effects were treated as random, a single variance for all position effects ${p}_{ijp}$ was fitted using a Toeplitz variance–covariance structure [58]. All analysis were performed using the PROC MIXED procedure of the SAS System Version 9.4 (Additional file 2).

### Evaluation criteria

For comparing the two arrangements, two measures of precision were calculated. Preferred arrangements will show a higher precision and therefore smaller values for the residual VC and the s.e.d. Note that the average s.e.d. is used as evaluation criterion, as the interest is usually to compare treatments. Estimating the differences of a pair of treatment estimates as precisely as possible occurs when the average standard error of these differences is minimized. The average s.e.d. and average residual VC within an arrangement was averaged across randomizations. Furthermore, the average VC for arrangement-specific residual variance was calculated. Afterwards, the ratio of average s.e.d. and VC for fixed-position designs to the corresponding value of re-arrangement was calculated. Furthermore, the usefulness of additional random incomplete block effects, positional effects, row and column effects and spatial correlation in (2) to (5) was checked by comparing AIC values of these models with the AIC value of model (1) [59].

## Supplementary information

**Additional file 1: Table S1.** Ratio of average error variance component (VC) compared to error VC under re-arrangement and average absolute error VC (in parenthesis) for seven traits and across traits under fixed-position arrangement using 15 different experimental designs across 200 randomizations. **Table S2.** Ratio of average s.e.d. compared to s.e.d. under re-arrangement and average absolute s.e.d. (in parenthesis) for seven traits and across traits under fixed-position arrangement using 15 different experimental designs across 200 randomizations. **Figure S1.** Picture of the greenhouse during germination. The picture is made from the northern side of the greenhouse showing the inner part of the greenhouse with the four tables and the mesh-protected outer part of the greenhouse in the south. **Figure S2.** Layout of the greenhouse showing the entry door in the north and two doors to the outer mesh-protected room in the south. Red rectangles represent tables with pots kept on fixed positions. Blue rectangles represent tables where pots were re-arranged. White rectangles are tables not used in the experiment.

**Additional file 2.** Data and complete program code written in SAS for performing simulations and analysis.

## Data Availability

The authors declare that all data and program code supporting the findings of this study are available within the additional information files (Additional file 2).

## Abbreviations

AIC: Akaike information criterion
C: carbon
N: nitrogen
RCBD: randomized complete block design
s.e.d.: standard error of a treatment difference
SPAD: single photon avalanche diode
VC: variance component
