# Supplementary material for: Blocking and re-arrangement of pots in greenhouse experiments: which approach is more effective?
Source: Plant Methods. 2019 Nov 27;15:143. doi: 10.1186/s13007-019-0527-4 (PMC6882062; doi:10.1186/s13007-019-0527-4)
Supplement: Supplementary file 1 — Additional file 1: Table S1. Ratio of average error variance component (VC) compared to error VC under re-arrangement and average absolute error VC (in parenthesis) for seven traits and across traits under fixed-position arrangement using 15 different experimental designs across 200 randomizations. Table S2. Ratio of average s.e.d. compared to s.e.d. under re-arrangement and average absolute s.e.d. (in parenthesis) for seven traits and across traits under fixed-position arrangement using 15 different experimental designs across 200 randomizations. Figure S1. Picture of the greenhouse during germination. The picture is made from the northern side of the greenhouse showing the inner part of the greenhouse with the four tables and the mesh-protected outer part of the greenhouse in the south. Figure S2. Layout of the greenhouse showing the entry door in the north and two doors to the outer mesh-protected room in the south. Red rectangles represent tables with pots kept on fixed positions. Blue rectangles represent tables where pots were re-arranged. White rectangles are tables not used in the experiment. [file 13007_2019_527_MOESM1_ESM.docx]

Table S1: Ratio of average error variance component (VC) compared to error VC under re-arrangement and average absolute error VC (in parenthesis) for seven traits and across traits under fixed-position arrangement using 15 different experimental designs across 200 randomizations. No values are calculated in case of spatial model, as error VC are not directly comparable. RCBD=randomized complete block design; SPAD= single photon avalanche diode.

| Design | | Ratio of error VCs§ (absolute error VC for fixed-position) for the traits | | | | | | | Average ratio^$^ of error VC across traits |
| --- | --- | --- | --- | --- | --- | --- | --- | --- | --- |
|  |  | Fresh weight | Dry weight | SPAD day 30 | SPAD day 36 | C content | N content | C to N ratio |  |
| Observed data | | 2.35  (34.92) | 1.33  (0.25) | 0.76  (14.79) | 0.76  (6.18) | 0.39  (0.069) | 0.99  (0.011) | 1.06  (0.011) | 1.09 |
| RCBD 1$\times$20  (1A) | | 2.40  (35.87) | 1.33  (0.25) | 0.74  (14.53) | 0.74  (6.08) | 0.39  (0.069) | 1.00  (0.011) | 1.04  (0.011) | 1.09 |
| α-design | 2$\times$10  (1B) | 2.19  (32.77) | 1.27  (0.24) | 0.74  (14.42) | 0.64  (5.30) | 0.35  (0.061) | 1.04  (0.011) | 1.07  (0.012) | 1.05 |
|  | 2$\times$10  (1C) | 1.64  (24.54) | 0.84  (0.16) | 0.73  (14.20) | 0.73  (6.06) | 0.38  (0.067) | 0.91  (0.010) | 1.01  (0.011) | 0.89 |
|  | $4\times$5  (1D) | 1.38  (20.55) | 0.76  (0.15) | 0.72  (14.02) | 0.65  (5.35) | 0.34  (0.059) | 0.87  (0.009) | 0.91  (0.010) | 0.81 |
|  | 4$\times$5  (1E) | 1.39  (20.71) | 0.63  (0.12) | 0.75  (14.62) | 0.74  (6.10) | 0.34  (0.060) | 0.80  (0.008) | 0.91  (0.009) | 0.78 |
|  | 4$\times$5  (1F) | 1.42  (21.25) | 0.70  (0.13) | 0.69  (13.49) | 0.74  (6.15) | 0.29  (0.051) | 0.77  (0.008) | 0.74  (0.007) | 0.75 |
|  | 5$\times$5  (1G) | 1.23  (18.43) | 0.59  (0.11) | 0.72  (13.96) | 0.72  (5.94) | 0.30  (0.053) | 0.68  (0.007) | 0.67  (0.007) | 0.70 |
|  | 5$\times$5  (1H) | 1.55  (23.10) | 0.79  (0.15) | 0.61  (11.94) | 0.67  (5.50) | 0.28  (0.050) | 0.79  (0.008) | 0.77  (0.008) | 0.78 |
|  | 5$\times$4  (1I) | 1.19  (17.71) | 0.65  (0.12) | 0.74  (14.47) | 0.66  (5.47) | 0.33  (0.059) | 0.68  (0.007) | 0.74  (0.008) | 0.71 |
|  | 5$\times$4  (1J) | 0.84  (12.57) | 0.60  (0.11) | 0.73  (14.30) | 0.67  (5.50) | 0.27  (0.047) | 0.70  (0.007) | 0.70  (0.007) | 0.63 |
|  | 10$\times$2  (1K) | 0.85  (12.69) | 0.54  (0.10) | 0.69  (13.48) | 0.62  (5.11) | 0.25  (0.044) | 0.71  (0.007) | 0.65  (0.007) | 0.62 |
|  | 10$\times$2  (1L) | 1.32  (19.66) | 0.59  (0.11) | 0.73  (14.28) | 0.68  (5.62) | 0.29  (0.052) | 0.46  (0.005) | 0.54  (0.006) | 0.66 |
| Row-column design | 2$\times$10  (1M) | 1.09  (16.30) | 0.51  (0.10) | 0.69  (13.56) | 0.56  (4.60) | 0.22  (0.038) | 0.55  (0.005) | 0.59  (0.006) | 0.59 |
|  | 2$\times$10  (1N) | 1.23  (18.36) | 0.54  (0.10) | 0.67  (13.09) | 0.53  (4.39) | 0.25  (0.043) | 0.44  (0.005) | 0.53  (0.006) | 0.60 |

^$^ Ratio of designs dependent error VC for fixed-position arrangement and error VC under re-arrangement

Table S2: Ratio of average s.e.d. compared to s.e.d. under re-arrangement and average absolute s.e.d. (in parenthesis) for seven traits and across traits under fixed-position arrangement using 15 different experimental designs across 200 randomizations. RCBD=randomized complete block design; SPAD= single photon avalanche diode.

| Design | | Ratio of average s.e.d.§ (absolute average s.e.d. for fixed-position) for the traits | | | | | | | Average ratio^$^ of s.e.d. across traits |
| --- | --- | --- | --- | --- | --- | --- | --- | --- | --- |
|  |  | Fresh weight | Dry weight | SPAD day 30 | SPAD day 36 | C content | N content | C to N ratio |  |
| RCBD 1$\times$20  (1A) | | 1.55  (5.96) | 1.15  (0.500) | 0.86  (3.80) | 0.84  (2.45) | 0.60  (0.262) | 0.95  (0.102) | 1.02  (0.104) | 1.00 |
| α-Design | 2$\times$10  (1B) | 1.54  (5.94) | 1.16  (0.504) | 0.88  (3.86) | 0.83  (2.42) | 0.60  (0.260) | 1.04  (0.112) | 1.10  (0.113) | 1.02 |
|  | 2$\times$10  (1C) | 1.37  (5.27) | 0.98  (0.425) | 0.87  (3.84) | 0.85  (2.48) | 0.61  (0.264) | 0.96  (0.103) | 1.06  (0.108) | 0.96 |
|  | $4\times$5  (1D) | 1.37  (5.27) | 1.02  (0.444) | 0.88  (3.89) | 0.85  (2.49) | 0.60  (0.263) | 0.99  (0.106) | 1.04  (0.107) | 0.97 |
|  | 4$\times$5  (1E) | 1.37  (5.27) | 0.94  (0.406) | 0.89  (3.92) | 0.86  (2.50) | 0.59  (0.259) | 0.94  (0.101) | 1.01  (0.103) | 0.94 |
|  | 4$\times$5  (1F) | 1.39  (5.34) | 0.98  (0.427) | 0.89  (3.91) | 0.87  (2.54) | 0.58  (0.253) | 0.93  (0.100) | 0.96  (0.098) | 0.94 |
|  | 5$\times$5  (1G) | 1.36  (5.24) | 0.95  (0.413) | 0.89  (3.90) | 0.85  (2.48) | 0.60  (0.261) | 0.93  (0.100) | 0.97  (0.099) | 0.94 |
|  | 5$\times$5  (1H) | 1.47  (5.64) | 1.07  (0.464) | 0.89  (3.91) | 0.87  (2.54) | 0.59  (0.258) | 0.97  (0.103) | 1.01  (0.103) | 0.98 |
|  | 5$\times$4  (1I) | 1.33  (5.12) | 0.99  (0.430) | 0.89  (3.90) | 0.85  (2.50) | 0.62  (0.269) | 0.93  (0.100) | 1.00  (0.102) | 0.94 |
|  | 5$\times$4  (1J) | 1.15  (4.14) | 0.96  (0.416) | 0.89  (3.92) | 0.86  (2.53) | 0.59  (0.255) | 0.93  (0.099) | 0.97  (0.099) | 0.91 |
|  | 10$\times$2  (1K) | 1.35  (5.17) | 1.05  (0.456) | 0.89  (3.93) | 0.87  (2.56) | 0.61  (0.265) | 0.97  (0.104) | 1.01  (0.103) | 0.96 |
|  | 10$\times$2  (1L) | 1.52  (5.85) | 1.09  (0.473) | 0.89  (3.91) | 0.86  (2.52) | 0.61  (0.267) | 0.92  (0.099) | 1.00  (0.102) | 0.98 |
| Row-column design | 2$\times$10 (1M) | 1.55  (5.94) | 1.13  (0.492) | 0.89  (3.91) | 0.85  (2.48) | 0.61  (0.266) | 1.04  (0.111) | 1.11  (0.113) | 1.02 |
|  | 2$\times$10 (1N) | 1.63  (6.25) | 1.16  (0.502) | 0.88  (3.87) | 0.83  (2.43) | 0.63  (0.274) | 0.94  (0.100) | 1.05  (0.107) | 1.02 |
| α-design (1B) with spatial error structure ^§^ | | 1.43  (5.51) | 1.10  (0.476) | 0.93  (4.10) | 0.86  (2.50) | 0.62  (0.271) | 1.02  (0.109) | 1.07  (0.109) | 1.00 |

^§^ Nugget variance plus a first order autoregressive error structure within a block of ten pots arranged in ten rows (Figure 1B)

^$^ Ratio of designs dependent s.e.d. for fixed-position arrangement and s.e.d. under re-arrangement

Figure S1

Figure S2
